# Prostate tumor markers: diagnosis, prognosis and management

**DOI:** 10.1590/1678-4685-GMB-2023-0136

**Published:** 2024-02-26

**Authors:** Gabriela Kniphoff da Silva Lawisch, Geórgia Muccillo Dexheimer, Vanderlei Biolchi, Rafael Armando Seewald, José Artur Bogo Chies

**Affiliations:** 1 Universidade do Vale do Taquari (Univates), Lajeado, RS, Brasil.; 2 Universidade Federal do Rio Grande do Sul, Departamento de Genética, Programa de Pós-Graduação em Genética e Biologia Molecular, Porto Alegre, RS, Brasil.; 3 Hospital Bruno Born, Centro de Oncologia, Lajeado, RS, Brasil.

**Keywords:** Prostate cancer, biomarkers, diagnosis, PSA, prognosis

## Abstract

Prostate cancer (PCA) is the second most common type of cancer in the world. Nevertheless, diagnosis is still based on nonspecific methods, or invasive methods which makes clinical decision and diagnosis difficult, generating risk of both underdiagnosis and overdiagnosis. Given the high prevalence, morbidity and mortality of PCA, new strategies are needed for its diagnosis. A review of the literature on available biomarkers for PCA was performed, using the following terms: prostate cancer AND marker OR biomarker. The search was carried out in Pubmed, Science Direct, Web of Science and Clinical Trial. A total of 35 articles were used, and PHI (Prostate Health Index) and the 4Kscore tests were identified as the best well-established serum markers. These tests are based on the evaluation of expression levels of several molecules. For analysis of urine samples, Progensa, ExoDXProstate, and Mi Prostate Score Urine Test are available. All these tests have the potential to help diagnosis, avoiding unnecessary biopsies, but they are used only in association with digital rectal examination and PSA level data. The search for biomarkers that can help in the diagnosis and therapeutic management of PCA is still in its initial phase, requiring more efforts for an effective clinical application.

## Introduction

Prostate cancer (PCA) is the most common type of cancer in men in the United States, excluding skin cancer. In 2023, the estimated number of new cases was 288,300, with an estimated mortality of 34,700 (American Cancer Society - http://www.cancer.org/cancer/prostate-cancer.html). In Brazil, 71,730 new PCA cases were estimated for each year in the three-year period 2023 - 2025 (Instituto Nacional de Cancer - http://www.inca.gov.br). PCA is one of the main causes of morbidity and mortality in men, being the second most common type of cancer and the fifth leading cause of cancer death worldwide, reaching 1.41 million cases and 370,000 deaths in 2020. It is also the most frequently diagnosed cancer in men, in more than half of the world’s countries (World Health Organization - http://www.who.int/news-room/fact-sheets/detail/cancer).

The early diagnosis of PCA, as well as other types of cancer, is related with a greater overall survival of patients. When diagnosis is achieved in the advanced stage, there is a significant reduction in patient survival. Also, the early detection of the tumor may represent less implications for the patient, slowing down the number of procedures and medications used, adverse events related to the disease and medications and, consequently, reflecting on a better quality of life (Resnick et al., 2013). Cancer is a heterogeneous pathology, with molecular variability that affects the response to treatment and the clinical course of the disease. Therefore, tools that allow optimizing patient care, such as markers for early diagnosis and therapeutic targets that allow efficient treatment are widely sought and studied (Cucchiara et al., 2018).

PSA is a protein produced by prostate cells, and elevated blood levels where frequently measured in the presence of prostate disease. Nevertheless, as the name implies, it is a prostate specific marker, not a cancer specific molecule, and may present high levels in cases of prostate hyperplasia and prostatitis. PSA assessment can bring many benefits in PCA screening, as due to earlier diagnosis, it can reduce the risk of developing metastases (Hugosson et al., 2019). However, since it is not cancer specific, this marker evaluation can be associated with unwanted effects, such as anxiety when faced with a false-positive result or even performing unnecessary biopsies, which also present inherent risks, such as infections and bleeding. These situations could negatively affect the patient’s quality of life (Carlsson and Vickers, 2020).

Currently, according to the National Comprehensive Cancer Network (NCCN), for healthy men, an age of 40 to 45 years is recommended for starting PCA screening, and continuing this procedure until the age of 75. This investigation is carried out in individuals over 75 years only in recommended cases (Carroll *et al*., 2019). In this scenario, there are cut-off points for the periodicity of PSA evaluation, since it not only helps in diagnosis, but also indicates a future risk. Therefore, for PSA values ​​below 1 ng/mL, a periodicity of 8 to 10 years can be adopted, from 1 to 2.99 ng/mL, the periodicity must be from 2 to 4 years and above 3 ng/mL, the patient should be monitored more carefully and perform additional tests (Kovac et al., 2020). 

Considering that symptoms are not easily observed on early prostate cancers, most cases are diagnosed by screening tests. The first evaluation includes a digital rectal exam (DRE) and PSA blood test, but the actual diagnosis of PCA can only be made with a prostate biopsy (ACS, 2023; NCCN, 2023). In order to reduce unnecessary biopsies and cases of overdiagnosis, and considering the limitations of DRE analysis (its sensitivity depends on the expertise of the physician, and if there is a large amount of inter-observer variability), an alternative is to perform magnetic resonance imaging (MRI) in patients with high levels of PSA. Although, it is still necessary to standardize this conduct, since biopsies performed only from an MRI result could lead to the non-detection of intermediate-risk tumors (Hugosson et al., 2022).

However, disease risk stratification and therapeutic decisions based only on PSA levels and prostate biopsy yield inaccurate results, which brings us back to the problems of overdiagnosis and overtreatment, in addition to interfering with the costs of health systems, which highlights the urgency of the search for new biomarkers. In this sense, currently, we still need to find specific molecular markers of PCA. The morbidity of the prostate biopsy exam and the treatments for this pathology (mainly radiotherapy and radical prostatectomy) make it necessary to establish more effective methods not only for the diagnosis of malignant neoplasm of the prostate, but also for the more accurate selection of the tumors that really are clinically relevant, that is, that can lead to patients death and effectively require more aggressive approaches. In this sense, this paper aimed to review the literature in order to describe the biomarkers currently used or potential for diagnosis, prognosis and management of PCA.

## Method

The literature on the subject of prostate cancer and biomarkers in different tissues was reviewed in order to identify tests that are already standardized and potential targets aiding the diagnosis, prognosis and clinical management of patients with suspected prostate pathologies.

The Scoping Review was performed using the following search terms: prostate cancer AND marker OR biomarker AND diagnosis. The search was performed in Pubmed, Science Direct, Web of Science and ClinicalTrial databases. The filter included free full articles published in the last 10 years (2012 to 2022). Review papers, unpublished data, comments, letters to the editor and responses to previous publications were excluded from the search. A total of 35 articles encompassing the research topic were obtained and used in this review.

## Results

**Figure 1 -:**
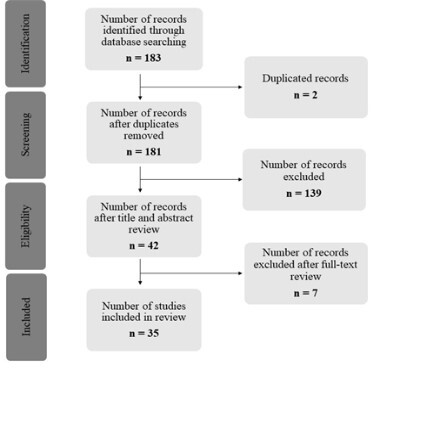
Flow chart of the selection of reviewed articles. 183 articles were found in database searching, and 2 were excluded for being duplicated. Then, 139 studies were excluded after analyzing the title and abstract, as they did not comply with the established inclusion criteria. Finally, all 42 articles were read in full, and 7 were excluded because they were reviews, letters to the editor, or responses to previous publications, resulting in a final number of 35 articles used in the present review.

A total of 183 articles were found in the search, and from these, 35 were included in this study after peer review by reading the abstracts and, later, full text articles (Figure 1).

### Sample biomarkers obtained by non-invasive techniques

Prostate biomarker research includes different classes such as DNA sequence changes, epigenetic changes (such as DNA methylation), gene expression changes, and protein markers. Biological samples used in the search for such markers can include blood, prostate tissue and urine. Some biomarkers are already being used in some countries to aid in diagnosis and prognosis without biopsy, or, in case of need for biopsy for confirmation, biomarkers contribute to the decision of therapeutic management, although there is still no global standardization for their use (Uhr et al., 2020).

Table 1 summarizes the main biomarkers described for assessing the diagnosis and prognosis of PCA.

**Table 1 -:** The main biomarkers described for PCA diagnosis and prognosis.

Assay	Sample	Biomarker	Advantages	Disadvantages	Reference
**PCA screening - tests that help in diagnosis and biopsy decision**					
Prostatic Specific Antigen	Serum	Total PSA.	Screening of man without symptoms.	Low specificity and does not indicate aggressiveness.	Carroll *et al*., 2019; Hugosson et al., 2019; Kovac et al., 2020.
Prostate Health Index (PHI)	Serum	Total PSA, free PSA (fPSA) and p2PSA.	Helps in clinical decisions (avoid unnecessary biopsies).	Different cutoffs described (mainly in different ethnic groups).	Perdona et al., 2013; Barisiene et al., 2020; Fan et al., 2021.
4KScore	Serum	Total PSA, fPSA, intact PSA and hK2.	Indicated for patients with altered PSA or DRE. Identify high risk cases, and help in biopsy decisions.	Values can be altered due to different individual clinical conditions.	Paerkh *et al*., 2015; Rannikko et al., 2022; Assel et al., 2019.
Filamin A, Filamin B, Keratin-19	Serum	FLNA, FLNB and KRT19.	Improved predictive power in detecting PCA (combined with PSA analysis).	The markers can be dysregulated in a variety of cancers, and need to be part of a panel.	Ravipaty et al., 2017.
Thrombospondin and D Cathepsin	Serum	Thrombospondin and D cathepsin.	Combined with free PSA, can improve PCA diagnosis and may reduce the number of unnecessary prostate biopsies.	Needs to be evaluated in different cohorts to establish a cutoff value.	Steuber et al., 2019.
PCA3	Urine	*PCA3*	Biopsy result prediction when combined with PSA and others.	The cutoff is not well established, there are cases with a high PCA3 value without PCA. Can’t be analyzed alone.	Wei et al., 2014; Fradet et al., 2018; Rubio-Briones et al., 2021.
ExoDXProstate	Urine	Exosomal mRNA for *PCA3, ERG* and *SPDEF*.	The test can discriminate high-grade from low-grade cancer and benign disease. And guides the biopsy-decision.	Should be used in combination with the PSA and other personal health factors.	McKiernan et al., 2018; Tutrone et al., 2020.
**PCA staging- tests that help establish the aggressiveness of the disease**					
MiPS	Urine	mRNA *PCA3* and *TMPRSS2:ERG.*	Diagnosis and prognosis with prediction of aggressiveness.	There are still no cutoff values determined.	Salami et al., 2013; Grupp et al., 2015; Tomlins et al., 2016; Alinezhad et al., 2016.
SelectMDX	Urine	mRNA *HOXC6, TDRD1* and *DLX1.*	Early diagnosis and predictor of aggressiveness (Gleason 7 or more).	No determined cutoff values, due to different values in distinct ethnic groups. Recommended before the biopsy decision.	Leyten et al., 2015; Hendriks et al., 2021.
ConfirmMDx	Tissue	DNA hypermethylation (*GSTP1, APC, RASSF1*)	Screens for prostate cancer at a molecular level, useful for patients who have a negative or inconclusive biopsy but there’s a high suspicion of PCA.	Accuracy and sensibility varies among studies, not FDA approved.	Wojno et al., 2014; Partin et al., 2014.
**PCA prognosis - tests that help to guide the treatment strategy**					
Prolaris	Tissue	Gene expression (31 genes).	Analyzes cell cycle progression score (predicts cancer aggressiveness, metastasis).	Needs an invasive sample, so can be done only after biopsy or prostatectomy.	Freedland et al., 2013; Cuzick et al., 2012.
OncotypeDx GPS	Tissue	Gene expression: *AZGP1, FAM13C, KLK2, SRD5A2, FLNC, GSN, GSTM2, TPM2, BGN, COL1A1, SFRP4, TPX2, ARF1, ATP5E, CLTC, GPS1, PGK1.*	Guides treatment (analyzes risk stratification) and aggressiveness (metastasis).	Only for organ confined PCA, and can be done after biopsy confirmation (invasive sample).	Van Den Eeden et al., 2018; Klein et al., 2014; Cullen et al., 2015.
Decipher	Tissue	Gene expression: *NFIB, NUSAP1, ZWILCH, ANO7, PCAT-32, UBE2C, CAMK2N1, MYBPC1, PBX1, THBS2, EPPK1, IQGAP3, LASP1, PCDH7, RABGAP1, GLYATL1P4, S1PR4, TNFRSF19, TSBP.*	Analyzes tumor aggressiveness, mortality and metastasis risk.	Only for organ confined PCA, and can be done after biopsy confirmation (invasive sample).	Gore et al., 2017; Cooperberg et al., 2015; Feng et al., 2021.
Promark	Tissue	Proteomics: DERL1, CUL2, SMAD4, PDSS2, HSPA9, FUS, pS6, YBOX1.	The test result is expressed as a score (from 0 to 100), with higher scores indicating a greater risk of aggressive disease.	The test is only meant to be performed on patients with biopsy Gleason specific scores, and needs a biopsy sample.	Shipitsin et al., 2014.
DNA-ploidy	Tissue	Assessment of DNA’s aberrant amount.	Prognostic marker in several cancer types. Patients with nondiploid tumors have an increased risk of poor prognosis compared to patients with diploid tumors.	General test, needs to be analyzed in combination with other biomarkers, and need a invasive sample.	Lennartz et al., 2016.
IMPROD bpMRI	Tissue	mRNA expression of ACSM1, AMACR, CACNA1D, DLX1, PCA3, PLA2G7, RHOU, SPINK1, SPON2, TMPRSS2-ERG, and TDRD1.	Diagnosis and analysis of aggressiveness.	The test is still being validated in different population groups.	Perez *et al*., 2020.

The biomarkers and tests recommended by the NCCN 2019 Guidelines include free PSA; Prostate Health Index (PHI), which uses total, free and p2PSA values and applies an algorithm to assess prostate health; 4Kscore, which evaluates the four PSA variants in the blood (total PSA, free PSA, intact PSA and human kallikrein 2 - hK2); and the ExoDxProstate (IntelliScore) which is a urine test that looks for RNA markers such as PCA3, *ERG* and *SPDEF* associated with high-grade PCA (Carroll *et al*., 2019). Nevertheless, it is important highlight that recommendations are updated frequently, and consequently the recommended tests could be changed. 

The possibility to use serum and/or urinary markers that indicate the presence of malignant alterations in the prostate allows the reduction of more invasive interventions, such as procedures for collecting tissue material for biopsies. This reflects in the patients’ better quality of life, reducing risks associated with the procedures, in addition to not generating anxiety and concern about procedures and possible diagnosis (Carlsson and Vickers, 2020).

The 4Kscore test is indicated for cases in which the serum total PSA dosage or DRE are altered. Thus, four markers are combined to predict the risk of PCA aggressiveness and, consequently, improve the assessment of the need for a biopsy. The evaluated markers are total PSA, free PSA, intact PSA and hK2. In this regard, total PSA and free PSA are general indicators for PCA with low specificity and do not represent the possible aggressiveness of the tumor. Intact PSA and hK2 are present at low concentrations in the bloodstream in healthy individuals and changes in these patterns are associated with aggressive types of cancer. These parameters, combined with the patient’s clinical data according to age and the presence of palpable nodules on DRE, are associated with an algorithm that indicates the possibility of a future prostate biopsy being positive with a Gleason greater than 7, that is, with higher risk of aggression. A study evaluating 1,012 patients, 231 with Gleason≥7, showed an excellent level of accuracy of 4Kscore in the discrimination between patients with high-risk disease who will benefit more from a biopsy compared to those with non-clinical significant tumor (Parekh et al., 2015; Eyrich et al., 2021). 

Among markers measured in serum, Filamin A, Filamin B and Keratin-19, are also validated as markers through ELISA techniques and mass spectrometry. It was demonstrated that the combination of these markers’ dosage with PSA was superior to the isolated use of PSA in PCA diagnosis, in addition to the prediction of a high or low degree of aggressiveness, and allowing a differentiation between cancer and benign prostatic hyperplasia (Ravipaty et al., 2017).

As previously mentioned, in addition to serological markers, good indicators of prostate health can also be obtained from urine samples, as biomarkers derived from the prostate and tumor cells from this tissue can be released into the prostate fluid and urine. Also, the urine obtained after prostatic manipulation is enriched with molecules released by the procedure, allowing the search for markers and malignant prostate cells, DNA, RNA, proteins and other small molecules (Fujita and Nonomura, 2018). In this sense, the oldest genetic biomarker in urine, PCA3 (Prostate Cancer gene 3), stands out. This gene has its high expression in PCA cell lines and prostatic tumors. The commercial test implemented in 2006 is called Progensa, and is considered by some authors to be superior to PSA analysis in the early detection of PCA. However, although this marker has good sensitivity, detecting small tumor volumes, it is not a good predictor of aggressiveness or indicator of tissue invasion. Thus, this marker is not indicated to help in prognosis, and is also insufficient to contraindicate a biopsy in cases of indolent cancer (Auprich et al., 2011).

Despite being a good marker, *PCA3* has limitations and has been studied in combination with other potential markers, in order to establish disease prediction algorithms. ExoDXProstate is a test that also helps in prostate biopsy decision and is measured in urine, being indicated for patients with PSA values between 2 and 10 ng/ml. This test evaluates the combined gene expression of *PCA3* and two other markers, *ERG* and *SPDEF*, generating a score that will be used in association with biopsy results (Tutrone et al., 2020). This test helps predict high-grade prostate cancer and can be applied as a marker of aggressiveness (McKiernan et al., 2018). With the same approach of markers associated with *PCA3*, MiPS (Mi Prostate Score Urine Test) stands out. This test evaluates, by multiplex technique, the *TMPRSS2:ERG* fusion gene and *PCA3*, associating them with serum levels of PSA. According to Salami et al. (2013), the combined use of these markers showed a better test’s predictive power for the presence of prostate cancer as compared to the same tests evaluated separately or when only the PSA serum level was evaluated. These markers help the early detection of prostate cancer. A study carried out with 1,225 patients observed that the application of this test combination improves the isolated serum PSA prostate cancer prediction performance, predicting not only the presence of PCA in the initial phase, but also indicating aggressive cancer. Thus, this combination is validated and can be used to estimate the individual risk (Tomlins et al., 2016).

Thrombospondin-1 (TSP-1) is a potent inhibitor of tumor angiogenesis and is important in controlling the malignant neoplasm’s growth (Vallbo and Damber, 2005). The regulation of *TSP* expression seems to be related with several pathways, including the *P53* tumor suppressor gene, and the inactivation of these genes seems to be related with a decrease in *TSP-1* expression and an increase in the angiogenic phenotype of the tumor (Su et al., 2010). Some studies have shown a decrease or even absence of this marker expression in cases of prostate tumor, while the expression keeps high in cases of prostatic hyperplasia and prostatic intraepithelial neoplasia (PIN) (Vallbo and Damber, 2005). Combinations of TSP-1 with other genes such as metalloproteinase-9 were evaluated, and a 2.8 times greater risk of developing prostate cancer was observed in patients with the combination of these markers. It has been shown that castration of rats with malignant prostate cancer induces the production of TSP-1 by the prostatic epithelium, while androgen replacement decreases its production. In humans, androgen deprivation decreased vascular endothelial growth factor (VEGF) expression and increased TSP-1 expression in human prostate carcinoma (Sfar et al., 2007).

A study conducted by Steuber et al. (2019) evaluated 476 men before performing a prostate biopsy, and analyzed serum thrombospondin and cathepsin D serum, combined with the percentage of free PSA. After taking into account results and outcomes, authors concluded that these analyses led to a better diagnosis of CaP, and reduced the number of unnecessary biopsies.

SelectMDx test evaluates *HOXC6, TDRD1* and *DLX1* gene expression. Leyten et al. (2015) conducted a gene expression study for a prostate cancer panel, evaluating 39 promising genes, and after a first analysis, selected eight genes. Afterwards, a combination of the expression of three genes in urine was indicated as a promising marker, as it presented greater accuracy in the prediction of aggressive prostate cancer with Gleason score greater than or equal to 7. This panel was also compared to the isolated evaluation of *PCA3*, and showed superior results. This panel proved to be efficient for identifying aggressive cancer even in patients with low serum PSA (Leyten *et al*., 2015).

More recently, Hendriks et al. (2021) analyzed 599 patients with SelectMDx and concluded that the test is able to avoid unnecessary biopsies, minimizes the detection of low-grade PCA and misses only 10% of high-grade PCA, in addition to having better results when used in combination with multiparametric magnetic resonance imaging of the prostate (image test capable of evaluating pathologies in the prostate region under different parameters: anatomy and prostatic morphology, cellularity, and vascularization of the lesion).

### Sample biomarkers obtained by invasive/tissue techniques

Although the search for non-invasive techniques is increasingly present and urgent, understanding the mechanisms involved in tumor progression is crucial for developing new strategies that could focus different processes, including minimizing the invasion of adjacent tissues and metastases. Therefore, the evaluation of markers in tissue material obtained by biopsy can also help in the diagnosis and prognosis. The marker Urokinase plasminogen activator (uPA) is known as an important component of tumor progression. uPA is highly expressed in malignant tumor cells and its activity is related to the conversion of plasminogen into plasmin, which acts in fibrinolysis regulation. Due to this activity, this molecule is able to hydrolyze components of the connective tissue of the matrix tissue. In addition, it has an activating action on metalloproteinase zymogens (MMPs). These enzymes hydrolyze components of the extracellular matrix, helping in the process of tumor invasion and metastasis. Therefore, uPA components can be used as diagnostic and prognostic markers for several types of cancer, also serving as therapeutic targets. The detection of high expression of *uPA*, *uPAR* (uPA-receptor) and *PAI-1* (plasminogen activator-1 inhibitor) can help in the clinical follow-up of the patient, intensifying the follow-up and evaluating possibilities of adjuvant therapy (Kimura et al., 2020).

Genetic analysis technology based on CRISPR/Cas9 has helped to understand mechanisms of tumor progression and therapeutic resistance. This, consequently, allowed the search for new genetic markers that could help the identification and development of new therapeutic targets. This technology has shown that the heterozygous deletion of 17p allows a selective dependence on *RBX1* (ubiquitin ligase E3 Ring-box 1) in castration-resistant metastatic PCA. *RBX1* activates *POLR2A*, elevating RNAP2-mediated mRNA synthesis. Thus, the combined inhibition of RNAP2 and *RBX1* suppresses tumor cell growth synergistically, improving the efficiency of the RNAP2 inhibitor conjugated antibody. Therefore, *RBX1* may be a therapeutic target for metastatic castration-resistant prostate cancer with heterozygous 17p deletion (Li et al., 2018).

The Prolaris test (Myriad Genetic Laboratories, Salt Lake City, UT) is a genetic panel that combines the evaluation of the expression of 31 genes, associated with cell cycle progression already identified as involved in the development of PCA. The sample used is formalin-fixed tissue collected for biopsy or radical prostatectomy. The result obtained after analyzing gene expression and applying algorithms can help not only to establish the patient’s prognosis, but also determine the intensity of follow-up for this patient and the indication for therapy (Cuzick et al., 2012; Freedland et al., 2013).

Oncotype Dx Genomic Prostate Score (GPS) (Genomic Health, Inc., Redwood City, CA), based on a RT-PCR technique, assesses gene expression of 12 genes associated with prostate cancer carcinogenesis. Such genes have effects on the induction of angiogenesis, cell proliferation and tissue organization. Tissue samples obtained by biopsy and already included in paraffin can be used, helping in their testing after an initial biopsy, at the time of diagnosis. After applying the genetic algorithm, scores are established for the patient’s sample, ranging from 0 to 100, the higher the number, the more aggressive the tumor, allowing the determination of the prognosis and determining the patient’s management, assessing the need for immediate treatment (Van Den Eeden et al., 2018).

Promark test (Metamark Genetics, Inc Cambridge, MA) analyzes eight proteins and allows a good prognosis assessment through algorithms indicative of tumor aggressiveness, in addition to helping in the therapeutic decision, and establishing the best management for each patient (Shipitsin et al., 2014).

DNA-ploidy is a cytogenetic evaluation test that allows the identification of the chromosome’s number. Using cytometry, the technique allows the indication of the DNA content in a cell, and the distribution of DNA among the cells of a tissue. Thus, its combination with the search for chromosomal deletions associated with the development of PCA may be clinically useful in the management of patients, which may indicate a worse prognosis (Lennartz et al., 2016). 

Regarding the therapeutic decision and patient management, the Decipher test is a commercial kit for the evaluation of gene expression and determines the risk stratification of newly diagnosed patients. This panel was applied in 150 patients referred for adjuvant radiotherapy and 115 patients referred for salvage radiotherapy after prostatectomy. After the gene expression analyses, there were changes in the management of some patients in both groups, improving the quality of each patient’s management (Gore et al., 2017).

### Liquid biopsy

Liquid biopsy consists of the real-time analysis of tumor cells or their products (such as free nucleic acids, vesicles or extracellular proteins) released into the blood or other body fluids, allowing the analysis of germline and somatic mutations, gene expression and signaling pathways. DNA and RNA circulating cellular fragments, which are released into the bloodstream, are stable for minutes or hours, and contain molecular alterations identical to the cells of origin, which allows the indirect analysis, in real time, of several genomic tumor alterations, such as mutations and rearrangements. Despite advances in the diagnosis and treatment of solid tumors, metastases remain the main cause of death in patients, and are more difficult to detect/diagnose. In addition, as mentioned earlier, the tumor is not homogeneous, making the analysis by tissue biopsy difficult, because it only accesses a small cell group, while liquid biopsy would have the ability to detect and analyze different cell types (Heidrich et al., 2020). Thus, liquid biopsy may have applicability in diagnosis, therapy, prognosis and patient follow-up.

A review by Ponti et al. (2021) addressed the analysis of free DNA and its fragmentations in liquid biopsies, showing that although there are differences in the quantifications performed in the different groups analyzed (PCA, benign prostatic hyperplasia, and controls), the methodologies used still differ greatly, generating different results, difficult to be compared.

Many of the biomarkers mentioned in Table 1 are also evaluated through liquid biopsy, making the analysis more specific, through the use of markers already well established in the literature, and also more accessible, as it is an easily obtainable sample (Matuszczak et al., 2021).

The number of publications about biomarkers assessed by liquid biopsy in PCA has increased dramatically in recent years, and there are several types of approach (there is an exponential increase in publications in Pubmed from 2012). Considering the differential diagnosis between PCA and benign prostatic hyperplasia, there is the advantage that other body fluids, in addition to serum or plasma, can be used with high sensitivity, such as urine and seminal fluid, due to their proximity to the prostate. Wang et al. (2021) demonstrated that liquid biopsy can bring important benefits in discovering mechanisms of resistance to treatment in patients with PCA, guiding therapeutic selection and early therapeutic switching during disease progression. Also, it has been indicated the possibility of assessing the aggressiveness of the disease through liquid biopsy (Ruiz-Plazas et al., 2021).

Currently, the ClinicalTrials.gov database (clinicaltrials.gov) registers 214 clinical trials with the terms “prostate cancer” and “circulating tumor cells”, 47 trials related to the term “circulating tumor DNA” and 24 related to “liquid biopsy”, reinforcing the potential of this methodology in studies involving prostate pathologies.

In its 2023 update, the NCCN recommends biomarker testing only for sporadic cancers, and only for individuals with metastatic disease, metastatic castration-resistant prostate cancer, patients who have had radical prostatectomy, or for patients in any stage of cancer but life expectancy greater than 10 years. However, the tests cited as most recommended (Decipher, Oncotype DX and Prolaris) evaluate tissue samples obtained by biopsy (NCCN, 2023). Despite advances in finding candidate genes for suitable biomarkers, few have been used in a clinical setting. That is, the search for biomarkers for PCA in non-invasive samples is still at an early stage. 

The assays PHI, 4Kscore test, PCA3 tests (such as Progensa) and ConfirmMDx have been recommended to help in biopsy decision, by identifying patients at low risk, where biopsy should be avoided (minimizing risks) and reducing the diagnosis of non-clinically significant tumors. Besides, the tests are also considered in cases where patients have a negative biopsy, but screening tests such as PSA and DRE are altered, which could raise the suspicion of a biopsy with a false negative result. That is, these tests have been used in the decision to perform a biopsy, or else in the evaluation of a new biopsy in case of suspicion of a false negative result. (ACS, 2023).

On the other hand, the new markers that analyze tissue, although they can help in prognosis, have the disadvantage of needing an invasive sample. In fact, they can also help in the treatment decision, since some patients have indications of active surveillance, while others may need definitive therapy, like radiotherapy or surgery. It is important to point out that there are also cases that need to undergo from the active surveillance to a therapy decision (NCCN, 2023).

Finally, the identification of PCA susceptibility genetic markers, as well as genetic markers associated with clinical outcomes, is another important field of research. In our laboratory, research has been carried out with important contributions in the field of biomarkers for prostate cancer and benign prostatic hyperplasia, such as associations with polymorphisms in the apoptosis genes *BCL-2*, *FASL* and *BAX* (da Silva Lawisch et al., 2022), *HLA-G* variant (Zambra et al., 2016) and polymorphisms in the *CCR2* and *CCR5* genes (Zambra *et al*., 2013).

## Final considerations

Considering the high age at the time of PCA diagnosis and the low aggressiveness of some tumors, the real indication for the use of more invasive procedures, such as biopsies or surgeries for radical prostatectomy should be better evaluated. In addition, the use of therapies for cancer treatment, such as radiotherapy or chemotherapy, must be evaluated taking into account the benefits against the possible risks. It should be noted that these therapies present numerous risks for the patient, with several adverse events reported, which may reflect on the patient’s quality of life, such as urinary or erectile dysfunction events, acute toxicities, leading to excessive hospitalization, and contributing to the increase of hospital expenses.

According to the data published in recent years regarding PCA incidence, it becomes evident that this pathology will continue to grow worldwide, and as a result, the search for more sensitive and specific biomarkers could help in early diagnosis and guide the best therapeutic approach, ensuring better chances of prognosis. Therefore, the search for PCA diagnosis and prognosis markers in minimally or non-invasive samples, such as serum and urine, should increase. Thus, it will be possible to avoid the use of invasive procedures for the collection of tissue material in patients who effectively do not have the need for such an evaluation. The analysis from tissue material still has the disadvantage that the tumor is not homogeneous, and the tissue biopsy restricts the analysis to the removed part. Also, in cases where serum markers indicate the need for tissue biopsy, the application of more complete tests, such as analysis of gene and/or protein expression mentioned above can contribute to better patient management, allowing the best therapeutic choice for each case. 
